# Educational Case: Rosai–Dorfman disease mimicking metastatic carcinoma

**DOI:** 10.1016/j.acpath.2025.100233

**Published:** 2026-01-02

**Authors:** Max Rogers, David T. Danielson, Ian Lagerstrom, Aaron Auerbach

**Affiliations:** a Department of Pathology, Walter Reed National Military Medical Center, Bethesda, MD, USA; b Joint Pathology Center, Silver Spring, MD, USA

**Keywords:** Pathology competencies, Diagnostic medicine, Surgical pathology, Differential diagnosis, Surgical pathology specimen derived from a lesion or mass, Histiocytosis, Rosai–Dorfman disease

The following fictional case is intended as a learning tool within the Pathology Competencies for Medical Education (PCME), a set of national standards for teaching pathology. These are divided into three basic competencies: Disease Mechanisms and Processes, Organ System Pathology, and Diagnostic Medicine and Therapeutic Pathology. For additional information, and a full list of learning objectives for all three competencies, see https://www.academicpathologyjournal.org/pcme.^1^

## Primary objective

Objective SP1.2: Differential Diagnosis. List the major differential diagnoses for a surgical pathology specimen obtained from a lesion or mass in a particular location and describe appropriate further studies to resolve the differential.^1^ Competency 3: Diagnostic Medicine and Therapeutic Pathology. Topic: Surgical pathology (SP). Learning Goal 1: Role in Diagnosis.

## Patient presentation

A 54-year-old woman with a past medical history of invasive breast carcinoma (IBC) of the right breast presents to her primary care provider for two months of low back pain. She describes the pain as dull, non-radiating, and worse when lying down. She denies fevers, chills, unintended weight loss, nausea, vomiting, recent infections, or other significant changes to her health. Her breast cancer was diagnosed two years ago, found to be positive for estrogen receptor and progesterone receptor biomarkers and negative for human epidermal growth factor receptor 2 (HER2), and was locally invasive without nodal metastases. She was treated with neoadjuvant chemotherapy followed by a lumpectomy with negative margins. She is not currently taking any medications and is unsure of her family medical history. During the visit, her vitals are notable for a temperature of 100.2 °F, heart rate of 78 bpm, blood pressure of 116/74 mmHg, and a respiratory rate of 12 breaths per minute. On physical exam, her lungs are clear to auscultation bilaterally without notable wheezes, rhonchi, or rales. Her heart has a regular rate and rhythm, with normal S1 and S2 heart sounds, and without any appreciable murmurs, rubs, or gallops. There are no deficits noted on a neurologic exam, and she has no spinal tenderness. However, she is noted to have non-tender, right axillary lymphadenopathy without any overlying skin changes.

## Diagnostic findings, Part 1

A complete blood count (CBC) is unremarkable. Magnetic resonance imaging (MRI) of the spine is obtained, with T1-weighted images revealing an irregular, hypoattenuating osteolytic lesion within the L4 spinous process.

## Questions/discussion points, Part 1

### What is the differential diagnosis for isolated lymphadenopathy?

Lymphadenopathy refers to the enlargement or altered consistency of lymph nodes.^2^ In adults, new-onset lymphadenopathy has a broad differential, ranging from reactive to malignant entities as well as primary nodal versus metastatic disease. A thorough history, physical exam, and in many instances, histologic evaluation, are essential to distinguish among the various possible etiologies. In this case, isolated right-sided axillary lymphadenopathy combined with a history of right-sided IBC raises concern for cancer recurrence with lymphatic spread. Other neoplastic etiologies, such as lymphoma or distant metastases, must also be considered, particularly if the patient exhibits “B symptoms” (fevers, night sweats, and weight loss).^2^^,^^3^ Even in the absence of these symptoms, axillary lymphadenopathy without an obvious infectious cause should prompt suspicion for malignancy.

While malignancy is high on our differential, benign causes of lymphadenopathy must also be considered (Table 1).^2^^,^^4^, ^5^, ^6^, ^7^ A variety of bacterial, viral, and fungal infections can lead to lymphadenopathy, necessitating correlation with the patient’s exposure history and clinical presentation.^2^ For instance, infection with *Bartonella henselae*, a Gram-negative rod, can cause “cat scratch disease”, which frequently presents with axillary lymphadenopathy.^2^^,^^8^ Infection with *Sporothrix schenckii* (sporotrichosis), a dimorphic fungus, often causes ascending or “sporotrichoid” lymphadenopathy.^2^^,^^9^ Human immunodeficiency virus (HIV) infections from both HIV1 and HIV2 can present with generalized lymphadenopathy, particularly in the cervical and axillary nodes, and should be suspected in patients with sexual histories that include multiple partners, condomless receptive vaginal or anal sex, and history of sex with a person that has an unknown HIV status or an unsuppressed HIV viral load.^2^^,^^7^ Additionally, infections from *Mycobacterium marinum*, an atypical or “non-tuberculosis” mycobacterium, may cause isolated lymphadenopathy with associated, overlying skin lesions in individuals that have been exposed to contaminated water sources.^10^

**Table 1:** Benign causes of lymphadenopathy.^2^^,^^4^, ^5^, ^6^, ^7^

Category	Causes
Infectious	- Viral: infectious mononucleosis (EBV), human immunodeficiency virus (HIV), Cytomegalovirus (CMV), varicella zoster virus, Herpes virus (HSV1/2), adenovirus, rubeola (measles), rubella - Bacterial: streptococcal pharyngitis, cat scratch disease, Cutaneous staphylococcal/streptococcal infections, chancroid, Tuberculosis, Atypical mycobacteria, Syphilis, tularemia, brucellosis, anaerobic dental infections - Fungal: histoplasmosis, aspergillus, blastomycosis, coccidioidomycosis, Sporotrichosis - Parasitic: toxoplasmosis, leishmaniasis - Other: Lyme disease (spirochete), rickettsial infections
Medications	allopurinol, phenytoin, carbamazepine, sulfonamides (e.g. trimethoprim-sulfamethoxazole), Penicillin, cephalosporin antibiotics, Hydralazine
Inflammatory	sarcoidosis, serum sickness, Kawasaki disease, Castleman disease, Crohn's disease, Kikuchi–Fujimoto disease, IgG4-related disease
Autoimmune	Systemic lupus erythematosus, Rheumatoid arthritis, Sjogren’s syndrome, dermatomyositis
Other	Histiocytosis, vasculitis

There are also many other noninfectious causes of lymphadenopathy. An autoimmune disease can lead to lymphadenopathy, though it may present as more diffuse rather than localized lymphadenopathy.^2^ Several medications, including penicillins, allopurinol, and hydralazine, as well as recent vaccinations, can cause transient lymphadenopathy. Lymphoproliferative disorders, such as Castleman disease, IgG4-related disease (IgG4-RD), histiocytic neoplasms, and causes of reactive lymphoid hyperplasia (e.g. dermatopathic lymphadenopathy) should also be considered. Given this patient’s history of IBC, imaging and tissue biopsy are the next logical steps to rule out metastatic disease.

### Interpret the MRI results. What is on the differential for an isolated lytic vertebral lesion? How does this factor in with our findings of axillary lymphadenopathy?

Osteolytic lesions, as observed in the MRI findings, refer to areas where normal bone has been replaced by tissue of a lower density, resulting in a sclerotic or “moth-eaten” appearance.^11^ The differential diagnosis for an osteolytic lesion is extensive, ranging from benign to malignant etiologies. For example, lesions with well-defined borders may suggest benign entities such as subchondral cysts, whereas poorly defined borders can indicate more aggressive pathology. These include inflammatory conditions like osteomyelitis and malignancies such as multiple myeloma and chondrosarcoma. Given this patient’s history of IBC, a metastatic disease should be strongly considered. Additionally, systemic diseases with variable clinical presentations, such as histiocytic disorders, should remain part of the differential.

### What are our next steps for diagnosis?

In light of the patient’s history, the presence of new axillary lymphadenopathy and an isolated, lytic vertebral lesion are highly suspicious for metastatic disease. The most appropriate next step is a comprehensive evaluation including a full-body positron emission tomography (PET)/computed tomography (CT) scan to assess for potential metastasis.

## Diagnostic findings, Part 2

**Fig. 1:**
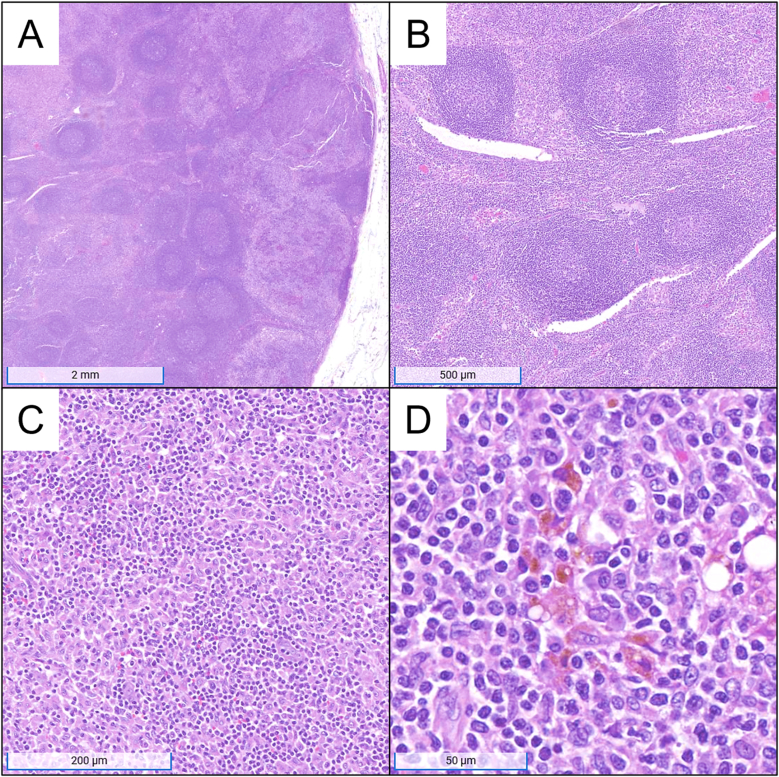
Representative microscopic images from the excisional lymph node biopsy. Panel A is a low power image showing a reactive-appearing lymph node with reactive follicular hyperplasia and sinus histiocytosis (hematoxylin and eosin; original magnification, 5x; scale bar, 2 mm). Panel B shows a higher power view of the interfollicular area, which is expanded by histiocytes (hematoxylin and eosin; original magnification, 20x; scale bar, 500 μm). Panel C is an intermediate power image of histiocytes mixed with small lymphocytes (hematoxylin and eosin; original magnification, 100x; scale bar, 200 μm). The histiocytes have characteristic features including variable nuclear shapes and abundant foamy, eosinophilic cytoplasms. Panel D is a high-power image showing melanin pigment in some of the histiocytes (hematoxylin and eosin; original magnification, 400x; scale bar, 50 μm).

The PET/CT scan reveals increased avidity in an enlarged right axillary lymph node and in the lytic vertebral lesion. An initial fine-needle aspiration (FNA) of the axillary lymph node is performed, but results are inconclusive due to inadequate tissue collection. Consequently, an excisional biopsy is conducted to provide a more definitive histopathologic evaluation. Representative microscopic images are provided in Fig. 1.

## Questions/discussion points, Part 2

### What does avidity indicate on PET scans? How does this influence management in cases of suspected malignancy?

Positron emission tomography/computed tomography scans utilize radiotracers, such as 18F-fluorodeoxyglucose (FDG), to identify metabolically active tissues throughout the body.^12^^,^^13^ Fluorodeoxyglucose, a glucose analog, is preferentially absorbed by cells with high metabolic demands, resulting in increased tracer uptake or avidity.^14^ When combined with concurrent CT imaging, there is a heightened ability to detect precise anatomic locations of avidity. While widely used to detect and monitor metastatic disease, FDG uptake is not exclusive to malignancy.^13^ Many reactive processes, including infections and inflammatory disorders such as sarcoidosis and granulomatous diseases, can also demonstrate avidity due to elevated metabolic requirements.^15^^,^^16^ Additionally, transient PET avidity has also been observed in axillary lymph nodes following certain vaccinations.^17^ Thus, while PET/CT scans are valuable for identifying potential malignancies, appropriate clinical and histologic correlation is essential to distinguish neoplastic processes from benign causes of avidity. In this patient’s case, concern for malignant spread warranted removal of axillary tissue for analysis. Given that the initial FNA results were inconclusive, an excisional lymph node biopsy was performed for comprehensive evaluation of nodal architecture and cellularity.

### Discuss and interpret the representative lymph node histology in Fig. 1. What features help delineate reactive lymphatic tissue from malignancy?

The tissue sampled displays a single encapsulated lymphatic lobule from the removed lymph node. Within the lobule, follicles are seen directly below the capsule in the subcapsular region or “superficial cortex.” The follicles have variation in their size and shape with an increased overall quantity. Mature, secondary follicles display pale-staining central areas known as germinal centers, which are surrounded by circular rims of dark, tightly packed cells referred to as mantle zones. Many of these follicles have enlarged germinal centers. On higher power, the germinal centers show a mix of centrocytes, centroblasts, follicular dendritic cells, and tingible body macrophages. Centrocytes are small lymphocytes with angulated nuclei and condensed chromatin. Centroblasts are large lymphocytes that have round-to-irregular nuclear borders, vesicular chromatin, and one or more peripherally located nucleoli. Tingible body macrophages are easily recognized due to their abundant pale cytoplasm with dark apoptotic bodies. A moderate amount of mitoses can be seen, and the mantle zone is sharply demarcated from each germinal center. Deeper to these follicles but superficial to the central medulla of the lobule is the paracortex. The paracortex in normal lymph nodes consists of predominantly small T-cells and dendritic cells. The paracortex in this case is expanded with an increased amount of dendritic cells, Langerhans cells, and macrophages, some of which contain dark, brown-staining melanin pigment. Sinus histiocytosis and mild capillary hyperplasia are present. Finally, there is no concern for involvement by metastatic breast cancer given an absence of atypical epithelioid cells.

This constellation of findings is most consistent with dermatopathic lymphadenopathy (DL). Dermatopathic lymphadenopathy is a benign cause of lymphadenopathy that is often associated with chronic dermatoses such as atopic dermatitis or psoriasis but can also be associated with neoplastic conditions including mycosis fungoides.^18^ It is histologically defined by reactive paracortical hyperplasia, seen in this case by a predominant paracortical expansion with a mixed cellular infiltrate and increased vascularity.^18^^,^^19^ Melanin-containing melanophages are also common in cases of DL but are not necessary for diagnosis. Reactive follicular hyperplasia is also observed and can be seen as a secondary process in around half of all cases of DL. As the reactive lymphatic processes here are hypermetabolic, they may present with PET avidity and mimic new malignancy or metastatic disease when found incidentally on routine imaging.^20^ The maximum standardized uptake value (SUVmax), a method of quantifying PET avidity, can be a sensitive tool for differentiating between metastatic and reactive lymph node processes in cases where a primary tumor is present.^21^

Though DL is most likely based on the histologic findings, it is necessary to rule out malignant processes. Follicular lymphoma should always be considered in cases of DL with follicular hyperplasia. Follicular lymphoma and reactive follicular hyperplasia will both have an increase in follicles on hematoxylin and eosin sections; however, in follicular lymphoma, the follicles may be more crowded with attenuated mantle zones.^20^^,^^22^ Moreover, the germinal centers in follicular lymphoma lack tingible body macrophages and germinal center polarity (a dark and light zone) will be absent. The majority of follicular lymphoma cases have a t(14; 18)(q31; q21) chromosomal translocation resulting in an *IGH::BCL2* fusion, leading to overexpression of the BCL-2 protein.^22^ BCL-2 is an antiapoptotic protein, which is not expressed in normal, nonneoplastic germinal center B-cells. Therefore, IHC staining for BCL-2 is often used in the diagnosis of follicular lymphoma as BCL-2 positivity in germinal center B-cells is present in most cases.^20^^,^^22^

Additionally, other malignant pathologies such as T-cell lymphomas and classic Hodgkin lymphoma need to be considered when paracortical hyperplasia is observed. T-cell lymphomas often demonstrate at least partially effaced normal lymph node architecture and cellular atypia within the paracortex, unlike the heterogeneous benign-appearing cellular infiltrate seen here. Hodgkin lymphoma can rarely present with an interfollicular growth pattern and often with increased numbers of histiocytes, which can lead to some histologic overlap with DL. However, classic Hodgkin lymphoma will have characteristic large neoplastic cells with pale cytoplasm, irregular nuclear contours, vesicular chromatin, and prominent nucleoli. The mononuclear variants of these cells are referred to as Hodgkin cells, while the multinuclear variant is referred to as Reed–Sternberg cells.^23^

Other benign, reactive processes that cause lymphadenopathy can have similar histologic findings and are important to consider. Isolated follicular hyperplasia can be a common finding in lymph nodes secondary to autoimmune diseases, like rheumatoid arthritis and Sjogren syndrome, as well as lymphoproliferative disorders such as Castleman disease.^19^ Lymphadenopathy secondary to infections, such as HIV, syphilis, and toxoplasmosis, also tend to have a predominately follicular pattern. Conversely, predominant paracortical hyperplasia can be associated with viral infections, including Epstein–Barr virus, cytomegalovirus, and herpes simplex virus. In the presented case, the histologic findings of prominent paracortical hyperplasia with increased dendritic cells, Langerhans cells, and histiocytes with occasional appreciable melanin pigment make the diagnosis of DL most likely. No viral cytopathic effects are seen to support a viral etiology.

### How is dermatopathic lymphadenopathy managed? What are the next diagnostic steps for this patient?

As previously mentioned, DL is a benign condition that is sometimes associated with serious dermatologic pathologies. When isolated and asymptomatic as in this case, DL is important to distinguish from primary malignancy or secondary metastases, thereby preventing the patient from undergoing further unnecessary or aggressive diagnostic work-up. Management in these instances is typically limited to periodic surveillance via imaging, and DL may spontaneously resolve after several months.^20^^,^^24^ However, this particular patient would not benefit from surveillance alone. Although her axillary lymphadenopathy was determined to be benign, the etiology of her lytic spinal lesion remains unclear, and it may still represent metastatic disease. Therefore, the appropriate next step in this case is to perform a biopsy from the lesion in the vertebra.

## Diagnostic findings, Part 3

**Fig. 2:**
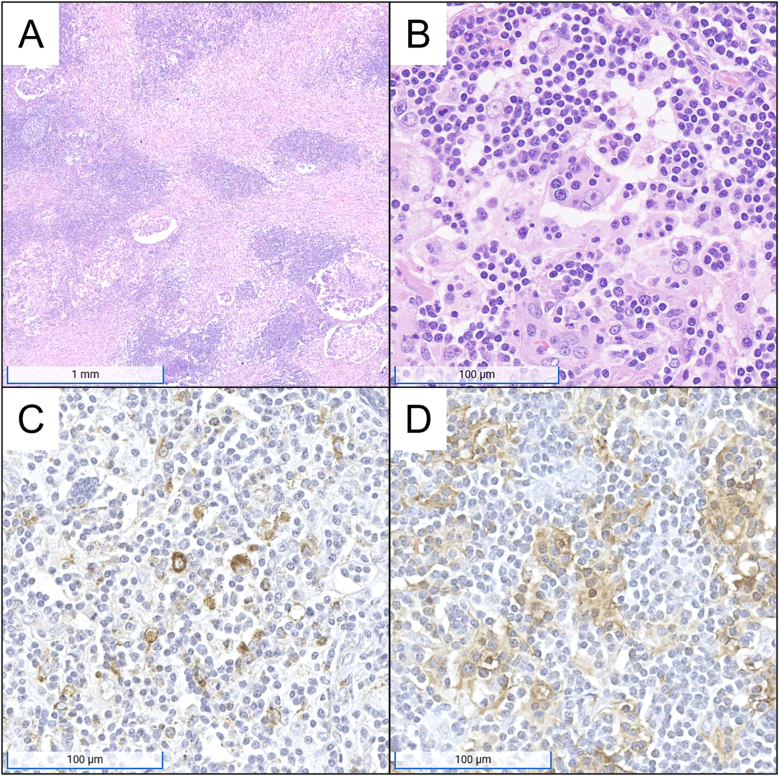
Representative microscopic images and immunohistochemical stains from the bone biopsy of the fourth lumbar vertebra. Panel A is a low-power image showing a diffuse infiltrate of histiocytes with associated fibrosis and mixed inflammatory cells (hematoxylin and eosin; original magnification, 10x; scale bar, 1 mm). Panel B is a high power image of the histiocytes, which display prominent emperipolesis (hematoxylin and eosin; original magnification, 200x; scale bar, 100 μm). Panel C is an immunohistochemical stain for CD68, which is immunoreactive in the histiocytes (original magnification, 200x; scale bar, 100 μm). Panel D is an immunohistochemical stain for S100, which is immunoreactive in the histiocytes (original magnification, 200x; scale bar, 100 μm). S100 is also useful to highlight emperipolesis as the engulfed inflammatory cells are not immunoreactive for S100.

A bone biopsy is taken from the lesion in the L4 vertebra. The specimen is submitted to surgical pathology for further evaluation. Representative microscopic images are shown in Fig. 2, panels A and B.

## Questions/discussion points, Part 3

### Describe the features seen on the slides in Fig. 2. Is there still concern for metastatic carcinoma?

The bone biopsy shows sheets of large pale cells admixed with fibrotic soft tissue. The cells exhibit round-to-oval nuclei with prominent nucleoli, and abundant eosinophilic cytoplasm that is foamy to finely granular. In some of these cells, there is evidence of smaller, intact inflammatory cells within the cytoplasm, a finding known as emperipolesis in this context. There are no atypical cells consistent with metastatic carcinoma.

### What is a histiocyte? How do the observed cell aggregates help narrow our differential?

Morphologically, the large, pale cells are histiocytes. Histiocytes are derived from myeloid precursors in the bone marrow and include macrophages and dendritic cells. In the context of a mass, the presence of many histiocytes raises concern for histiocytosis. Histiocytosis is a term that refers to a group of disorders characterized by a proliferation of histiocytes with overlapping features of inflammatory and neoplastic conditions.^25^ They can be idiopathic or secondary to diseases, such as malignancy or autoimmune disorders. Histiocytosis can involve many different body sites; therefore, clinical manifestations of histiocytosis vary widely.

### What are the histiocytosis subtypes? Between Langerhans cell histiocytosis, Erdheim–Chester disease, and Rosai–Dorfman disease, which histiocytosis are the patient’s findings most consistent with?

Historically, histiocytosis subtypes were defined by histologic features, and included (LCH), Erdheim–Chester disease (ECD), and Rosai–Dorfman disease (RDD).^25^^,^^26^ The most recent consensus classification now defines five subtypes based on multiple clinical, pathologic, and genetic features: the Langerhans-related (L), cutaneous and mucocutaneous (C), malignant (M), hemophagocytic lymphohistiocytosis and macrophage activation syndrome (H), and Rosai–Dorfman and miscellaneous non-cutaneous, non-Langerhans cell (R) groups.^26^ The L group of histiocytoses includes LCH and ECD based on common co-occurrence and similar genetic alterations. Langerhans cell histiocytosis is characterized by a proliferation of clonal Langerhans cells that primarily affects bone and skin, and ECD is a multisystem disease with bony, cardiovascular, and central nervous system involvement. Rosai–Dorfman disease, historically described as “sinus histiocytosis with massive lymphadenopathy,” is subcategorized into sporadic, familial, and cutaneous subtypes, with the former two residing within the R group.^26^^,^^27^ All three of these diseases can present as bony lesions and they cannot be diagnosed on radiographic imaging alone.

The aforementioned histologic findings are most consistent with RDD. Rosai–Dorfman disease is characterized by sheets of histiocytes with abundant pale cytoplasm, round nuclei, vesicular chromatin, and centrally located prominent nucleoli.^27^ The presence of emperipolesis, or trafficking of inflammatory cells through the cytoplasm of histiocytes without degradation, is a hallmark of RDD.^26^ Langerhans cell histiocytosis is less likely in this case given the presence of emperipolesis as well as an absence of classic Langerhans histiocyte cells with their characteristic grooved or “coffee bean” nuclei. Furthermore, though ECD may rarely show emperipolesis, it is lower on the differential as it typically demonstrates lipid-laden or “foamy” histiocytes, additional sparse multinucleated histiocytes known as “Touton giant cells”, and abundant background fibrosis.

With a higher suspicion for RDD, the location of the lesion helps specify the subtype. Classic nodal RDD manifests in lymph nodes and commonly presents as bilateral cervical lymphadenopathy with associated systemic symptoms in younger individuals.^28^ Conversely, extranodal RDD is more common in older adults and can involve multiple sites, including bone, skin, and nasal cavities. Due to this variability, extranodal RDD has a broad, nondescript range of clinical features and has presented in many cases, similar to this case, as isolated vertebral lesions with symptoms ranging from vague back pain to compressive neuropathy.^29^, ^30^, ^31^, ^32^

Extranodal RDD can appear less distinctive on biopsy compared to classic nodal RDD. This is typically secondary to increased fibrosis, fewer histiocytes, and less frequent emperipolesis in extranodal form.^27^^,^^28^ These findings can also be seen in some cases of LCH and ECD. Furthermore, RDD often has a similar histologic appearance to IgG4-RD, which may also present as an isolated mass. Although the findings so far favor extranodal RDD, histologic findings alone are not enough to form a diagnosis and immunophenotype characterization with IHC staining is required.

## Diagnostic findings, Part 4

Immunohistochemistry staining is performed on the bone marrow biopsy for numerous antigens, including CD1a, CD68, and S100. Representative images of the IHC staining for CD68 and S100 are shown in Fig. 2, panels C and D. The histiocytes are positive for CD68 and S100, as shown, and are negative for CD1a.

## Questions/discussion points, Part 4

### Interpret the provided IHC results. How do positive and negative IHC results guide our overall interpretation of the lesion?

Immunohistochemistry allows for more specific characterization of cells, requiring a panel of stains to diagnose RDD while excluding histologic mimics. First, the cells of interest are CD68-positive. CD68 positivity confirms the presence of histiocytes as CD68 is a marker of the monocyte/macrophage lineage. S100 positivity, commonly seen in RDD and LCH, reinforces the likelihood of a histiocytic disorder. CD1a negativity is a critical finding as it helps exclude LCH especially in combination with the absence of eosinophil-rich infiltrates and prominent nuclear grooves.^28^ In challenging cases, additional IHC markers are often required to differentiate RDD from other conditions.

### Rosai–Dorfman disease is commonly associated with gain-of-function mutations. What are gain-of-function mutations, and how could testing for these mutations be of clinical use for the patient?

Recent advances in genetic analysis, notably next-generation sequencing (NGS), have demonstrated that many histiocytoses, including RDD, are associated with gain-of-function mutations. These mutations affect genes responsible for cell proliferation and replication causing them to become constitutively active, which may drive neoplastic processes. One key example is *KRAS*, a cell-signaling gene within the mitogen-activated protein kinase pathway that promotes cellular growth. *KRAS* mutations, along with *MAP2K1*, are frequently found in RDD and likely contribute to its role as a clonal histiocytic neoplasm.^33^ By contrast, *BRAF* (V600E) mutations are much more common in LCH and ECD.^34^

Although most cases of RDD are self-limited or respond well to first-line treatments such as surgical excision, corticosteroids, sirolimus, and/or radiotherapy, some cases may present as multifocal, aggressive, or refractory disease.^27^ In these challenging cases, standard chemotherapy and immunomodulators such as rituximab have shown mixed efficacy. Targeted therapy that is guided by NGS findings has proven useful in these instances.

### What other evaluation should be performed for a patient that has been diagnosed with RDD?

As previously mentioned, RDD has a broad range of clinical manifestations and co-occurring disorders. Recommended baseline labs include a CBC with differential as RDD often presents with normocytic anemia, thrombocytopenia, and/or eosinophilia.^27^^,^^34^ If abnormalities are observed, a subsequent peripheral smear may be indicated. A comprehensive metabolic panel is also recommended to evaluate hepatic involvement, and erythrocyte sedimentation rate and C-reactive protein levels may be used to track the presence of an inflammatory process. If autoimmune diseases are suspected clinically, certain blood tests such as an antinuclear antibody, rheumatoid factor, and an autoimmune lymphoproliferative syndrome panel may also be warranted. If not already performed, full-body imaging via PET/CT is typically recommended for suspected histiocytosis to assess for other areas of involvement. As is seen in this case, RDD lesions demonstrate PET avidity that is often comparable to the expected avidity from certain malignancies, though additionally calculating the SUVmax for these areas may be helpful for differentiating between the two.^22^^,^^27^ Additionally, MRI can be used in pediatric cases, where full-body CT scans are less advisable, or for dedicated organ-specific screening.

## Teaching points

• The differential diagnosis for new lesions on imaging and lymphadenopathy is broad, even in patients with a history of malignancy. In this regard, benign/reactive etiologies should always be considered before anchoring a diagnosis on metastasis.
• Positron emission tomography scans demonstrate avidity based on the uptake of a radiotracer glucose analog, and abnormal uptake in tissues may represent either reactive or malignant processes.
• Isolated dermatopathic lymphadenopathy is a rare cause of benign lymphadenopathy, and histologic examination is necessary for both diagnosis and to rule out malignancies such as lymphoma or metastatic disease.
• The histiocytoses are a group of disorders that contain elements of both inflammatory and neoplastic diseases. These conditions can be difficult to diagnose because they may present with a broad range of clinical manifestations.
• Rosai–Dorfman disease (RDD) is a rare, heterogenous histiocytosis that arises in nodal and extranodal sites. Extranodal forms may present as isolated vertebral lesions, closely mimicking a metastatic disease in the appropriate clinical context.
• Although emperipolesis is a common finding in RDD, definitive diagnosis relies on immunohistochemical analysis. The presence of histiocytes that are S100+, CD68^+^, and CD1a help distinguish RDD from common lookalikes such as Langerhans cell histiocytosis.
• Next-generation sequencing has helped demonstrate clonality in cases of RDD and is a useful tool in diagnosis and for guiding treatment options in complicated cases.

## Author's note

The views expressed in this manuscript are those of the authors and do not necessarily represent the official policy of the Uniformed Services University of the Health Sciences (USUHS), Defense Health Agency, the Department of Defense (DOD) or the US Government.

## Funding

The article processing fee for this article was funded by an Open Access Award given by the Society of ‘67, which supports the mission of the Association for Academic Pathology to produce the next generation of outstanding investigators and educational scholars in the field of pathology. This award helps to promote the publication of high-quality original scholarship in *Academic Pathology* by authors at an early stage of academic development.

## Declaration of competing interest

The authors declare that they have no known competing financial interests or personal relationships that could have appeared to influence the work reported in this paper.
